# Metformin suppresses proliferation and glycolysis of gastric cancer by modulating ADAMTS12

**DOI:** 10.1186/s41021-023-00296-z

**Published:** 2024-01-03

**Authors:** Rui Chen, Jianhui Chen, Miaoliang Chen, Shenkang Zhou, Pinlu Jiang

**Affiliations:** 1 Department of Gastroenterology, Taizhou Hospital of Zhejiang Province, 317000 Taizhou, Zhejiang PR China; 2 Department of Emergency, Taizhou Hospital of Zhejiang Province, 150# Ximen Street, 317000 Taizhou, Zhejiang PR China

**Keywords:** Metformin, ADAMTS12, Gastric cancer, Cell proliferation, Glycolysis

## Abstract

**Background:**

Gastric cancer (GC) is a common malignancy with its morbidity increasing worldwide. Hence, it is imperative to develop effective treatments. Studies have shown that metformin has potential antitumor effects. The objective of this study was to probe the antitumor mechanism of metformin in GC.

**Methods:**

The expression of ADAMTS12 in GC tissues and its enrichment pathways were analyzed by bioinformatics methods. ADAMTS12 expression in GC cells was assessed by qRT-PCR. Cell viability and proliferation were analyzed by CCK-8 and colony formation assays, respectively. Extracellular acidification rate (ECAR) and oxygen consumption rate (OCR) of GC cells in different treatment groups were analyzed by Seahorse XP 96, and glycolysis metabolites were detected by corresponding kits. Western blot was employed to analyze the level of glycolysis pathway related protein HK-2, and cell functional assays were conducted to verify the functions of metformin on GC cells. A xenograft model was constructed to validate the inhibitory role of metformin in GC.

**Results:**

ADAMTS12 expression was elevated in GC tissues/cells and concentrated in glycolysis pathway. Cell functional assays found that ADAMTS12 promoted the proliferation and glycolysis of GC cells. Rescue experiments showed that metformin could reduce the promoting effect of ADAMTS12 overexpression on the proliferation and glycolysis of GC cells. In vivo studies confirmed that metformin suppressed the proliferation and glycolysis process via ADAMTS12 in GC cells.

**Conclusion:**

Metformin can repress the proliferation and glycolysis of GC cells via ADAMTS12. The results suggest the potential of ADAMTS12 being a target for the metformin therapy of GC.

## Introduction

Globally, gastric cancer (GC) is the fifth among cancer diagnoses and the fourth in cancer-related deaths [1]. Among GC cases, nearly half were Chinese, and the majority have been diagnosed in advanced stage of lymph node metastasis or distal metastasis. Despite development of treatment strategies, its prognosis remains poor [2]. Thus, it is of great importance for the development of effective therapies to further study the molecular mechanisms related to GC progression.

Even when oxygen is plentiful, cancer cells prioritize glycolysis over mitochondrial oxidative phosphorylation to generate glucose-dependent ATP and glucose oxidation intermediates, which is called aerobic glycolysis [3]. The important role of aerobic glycolysis in cancer has been widely studied, and the molecular mechanisms affecting glycolysis in GC cells have been reported in many studies. For example, SALL4 promotes GC progression through hexokinase II-mediated glycolysis [4]. Similarly, metabolic reprogramming can influence the tumor microenvironment, thus affecting tumor progression [5]. Xingxing Yao et al. [6] found that SLC2A3 facilitates the transition of macrophages to M2 subtype in the GC microenvironment. Hence, it is of great significance to elucidate the molecular mechanism of glycolysis in tumor cells for understanding the progression of GC.

ADAMTS12 is a member of the ADAMTS family [7]. Numerous previous studies have shown that ADAMTSs exhibit pro-tumor or antitumor roles in proteolytic and non-proteolytic forms. In renal cell carcinoma, the up-regulated ADAMST12 expression promotes tumor metastasis [8]. Conversely, ADAMST12 deficiency leads to an enhanced angiogenic response and increased tumor invasion, as demonstrated by in vitro and in vivo studies, suggesting that ADAMTS12 is a possible antitumor factor [9]. However, the specific function and mechanism of ADAMTS12 in glycolysis of GC cells have not been reported yet. Therefore, understanding the regulatory mechanism of ADAMTS12 in glycolysis of GC is of great value for GC management.

Metformin is considered to be the first-line therapy for type 2 diabetes. According to studies over the past 10 years, metformin has the potential to be used as an effective anticancer drug in a variety of tumors [10–12]. In non-small cell lung cancer, metformin inhibits cell growth via the AMPK/mTOR signaling pathway [13]. In ovarian cancer, metformin hinders the malignant progression of tumor cells by down-regulating the expression of MSLN and the activity of IL-6/STAT3 signaling pathway, showing an anticancer role [14]. Also, it has been shown that metformin represses cell proliferation, causes cell death, and leads to partial cell cycle arrest in GC [15]. In conclusion, numerous epidemiological investigations and clinical studies have revealed the different mechanisms of metformin against tumor. However, the exact effects of metformin on GC remain imperfect. Hence, this study will further explore the molecular mechanism by which metformin inhibits GC.

We analyzed the level and function of ADAMTS12 in GC, investigated the role of metformin in GC, and clarified the mechanism of metformin/ADAMTS12 affecting the proliferation and glycolysis of GC. As the results demonstrated, ADAMTS12 expression was significantly increased in GC tissues and cells, and ADAMTS12 overexpression facilitated the proliferation and glycolysis of GC cells. On the other side, metformin could down-regulate ADAMTS12 expression and hinder cell proliferation and glycolysis. In vivo experiments also demonstrated the inhibitory effect of metformin on proliferation and glycolysis of GC cells. In one word, our study revealed a potential target by which metformin plays antitumor effects in GC, providing new insights into the role of metformin in GC treatment.

## Materials and methods

### Bioinformatics

The mRNA data of GC were downloaded from The Cancer Genome Atlas (TCGA) database. The differentially expressed (DE) mRNAs were obtained by analysis with edgeR. The target gene was identified by combining bioinformatics data with literature and subjected to Gene Set Enrichment Analysis (GSEA).

### Cell culture

Human GC cell lines (AGS, HGC-27 and MGC-803) and gastric mucosal epithelial cell line (GES-1) were accessed from BeNa Culture Collection (BNCC, China). AGS cells were kept in F-12 K medium. HGC-27 cells and MGC-803 cells were maintained in RPMI-1640 medium. GES-1 cells were preserved in DMEM-H medium. All above mediums were from Gibco, USA and supplemented with 10% fetal bovine serum (FBS) as well as 1% penicillin-streptomycin. The FBS used in the experiments were from Tianjin Kang Yuan Biology, China, while the penicillin and streptomycin were from Thermo Fisher Scientific, USA. The culture conditions were 37 ℃ with 5% CO_2_ [16].

### Cell transfection

si-ADAMTS12, pcDNA 3.1-constructed oe-ADAMTS12, and the corresponding negative controls were accessed from Ribobio (China), and metformin, DMSO, and PBS were from Sigma (USA). Lipofectamine 3000 (Invitrogen) was utilized for AGS cell transfection with plasmids. 48 h after transfection, cells were used for functional assays [17].

### qRT-PCR

Total RNA was extracted using Trizol (TaKaRa, Japan). RNA was reversely transcribed to cDNA by the utilization of the PrimeScript™ RT Reagent Kit and gDNA Eraser (TaKaRa, Japan). qRT-PCR was conducted using TB Green® Fast qPCR Mix (TaKaRa, Japan). We adopted β-actin as the internal reference for ADAMTS12. The 2^−ΔΔCt^ means was employed to calculate relative expression of target gene. The experiment was done in triplicate. Primer sequences are listed in Table 1.

**Table 1:** Primers used in qRT-PCR

Gene	Sequence	
ADAMTS12	Forward Primer	5’-CGGGAGGAAGATGTATCGAGC-3’
	Reverse Primer	5’-TCAACTAACAATATCCGCTTTCG-3’
HK-2	Forward Primer	5’-GCCATCCTGCAACACTTAGGGCTTGAG-3’
	Reverse Primer	5’-GTGAGGATGTAGCTTGTAGAGGGTCCC-3’
Ki67	Forward Primer	5’-CGGAAGAGCTGAACAGCAACGA-3’
	Reverse Primer	5’-GCGTCTGGAGCGCAGGGATA-3’
β-Actin	Forward Primer	5’-CACGAAACTACCTTCAACTCC-3’
	Reverse Primer	5’-CATACTCCTGCTTGCTGATC-3’

### CCK-8

Regarding detection of cell viability, CCK-8 assay was recommended. The transfected cells (1 × 10^4^ cells/well) were plated with 96-well plates and 10 µL CCK-8 (Dojindo, Japan) was introduced at 0, 24, 48, and 72 h, separately. The absorbance at 450 nm was read using an enzyme marker after 2 h of incubation protected from light. To measure the sensitivity of cells to metformin, GC cells AGS were plated with 96-well plates and treated with different concentrations (0, 5, 10, 15, 20 mM) of metformin for 72 h. The CCK-8 solution of 10 µL was introduced for 2 h of incubation protected from light. The OD value at 450 nm was assessed by a microplate reader [18].

### Colony formation assay

The transfected cells (1 × 10^3^ cells/well) were seeded in 12-well plates. Mediums were replaced every 3 d for about 10 d. When most cell colonies contained more than 50 cells, they were immobilized with 4% paraformaldehyde, followed by staining with 0.1% crystal violet and counting.

### Western blot

Protein isolation was operated by using RIPA lysis buffer (pH 8.8, 50 mM Tris-HCl, 150 mM NaCl, 0.1% SDS, 1% sodium deoxycholate, 1% NP-40). The concentration of the proteins was assayed using the BCA Protein Assay Kit. The protein samples of 50 µg were separated on a 10% SDS-PAGE gel and blotted onto a PVDF membrane. The membrane was blocked with 5% skim milk in TBST buffer, followed by incubation with primary antibodies overnight at 4 ℃ and incubation with secondary antibody. The protein bands were assayed by using the Enhanced Chemiluminescence (ECL) assay kit (Yeasen, China). Primary antibodies rabbit anti-human Anti-HK-2 (ab209847), Anti-β-Actin (ab8226), Anti-ATP5D (ab97491), and secondary antibody goat anti-rabbit Anti-IgG (ab6721) were provided by Abcam (UK). Primary antibody rabbit anti-human Anti-ATP5E (PA5-104424) was provided by Thermo Fisher Scientific (USA).

### Extracellular acidification rate (ECAR) and oxygen consumption rate (OCR)

Cellular glycolysis and oxidative phosphorylation were analyzed using Glycolysis Cell-Based Assay Kit (Cayman, UK), Seahorse XF Cell Mitochondrial Stress Test Kit (Agilent, USA), and Seahorse XF96 Analyzer (Agilent, USA). 5 × 10^4^ cells were plated in XF 96 Cell Culture Microplate (Agilent, USA) and cultured for 10 h. The levels of ECAR and OCR were detected and analyzed using the Seahorse XF96 Extracellular Flux Analyzer (Agilent, USA). The specific steps were referred to a previous study [19].

### Detection of pyruvic acid, lactate, citrate, and malate levels

The assay kits were utilized to detect the production of glycolytic metabolites in cells, including pyruvic acid, lactate, citrate, and malate. Pyruvate Assay Kit (Catl. No. A081) and Citric acid (CA) content of the test box (Catl. No. A128) were from Nanjing Jiancheng Bioengineering Institute (China). Lactate Assay Kit (Catl. No. K951) and Malate Assay Kit (Catl No. K637) were purchased from BioVision (USA).

### Xenograft mouse model construction

Twenty BALB/c-nu mice were bought from Shanghai SLAC (China). All procedures were examined and approved by the Animal Ethics Committee. All animal studies were completed as per the ARRIVE guidelines. The mice were divided into four groups with five in each. GC cells stably transfected with oe-NC and oe-ADAMTS12 were injected subcutaneously into the mice. When the tumors grew to 100 mm^3^, metformin (250 mg/kg) or PBS was administered. After 7 d of treatment, the size of tumors was measured every 3 d with digital calipers. The volume of tumors was obtained using the formula: length × width^2^ × 0.5. The mice were euthanized after 25 d. Tumors were excised, photographed, and weighed, and tumor tissues were collected for analysis [20].

### Statistics

Data were expressed in the form of mean ± standard deviation (SD). GraphPad 8.0 was employed for statistical analysis. T-test was utilized for comparisons between two groups, while univariate analysis for those between multiple groups. *P*-values below 0.05 were defined as having a significant level.

## Results

### ADAMTS12 is highly expressed in GC tissues and cells

**Fig. 1:**
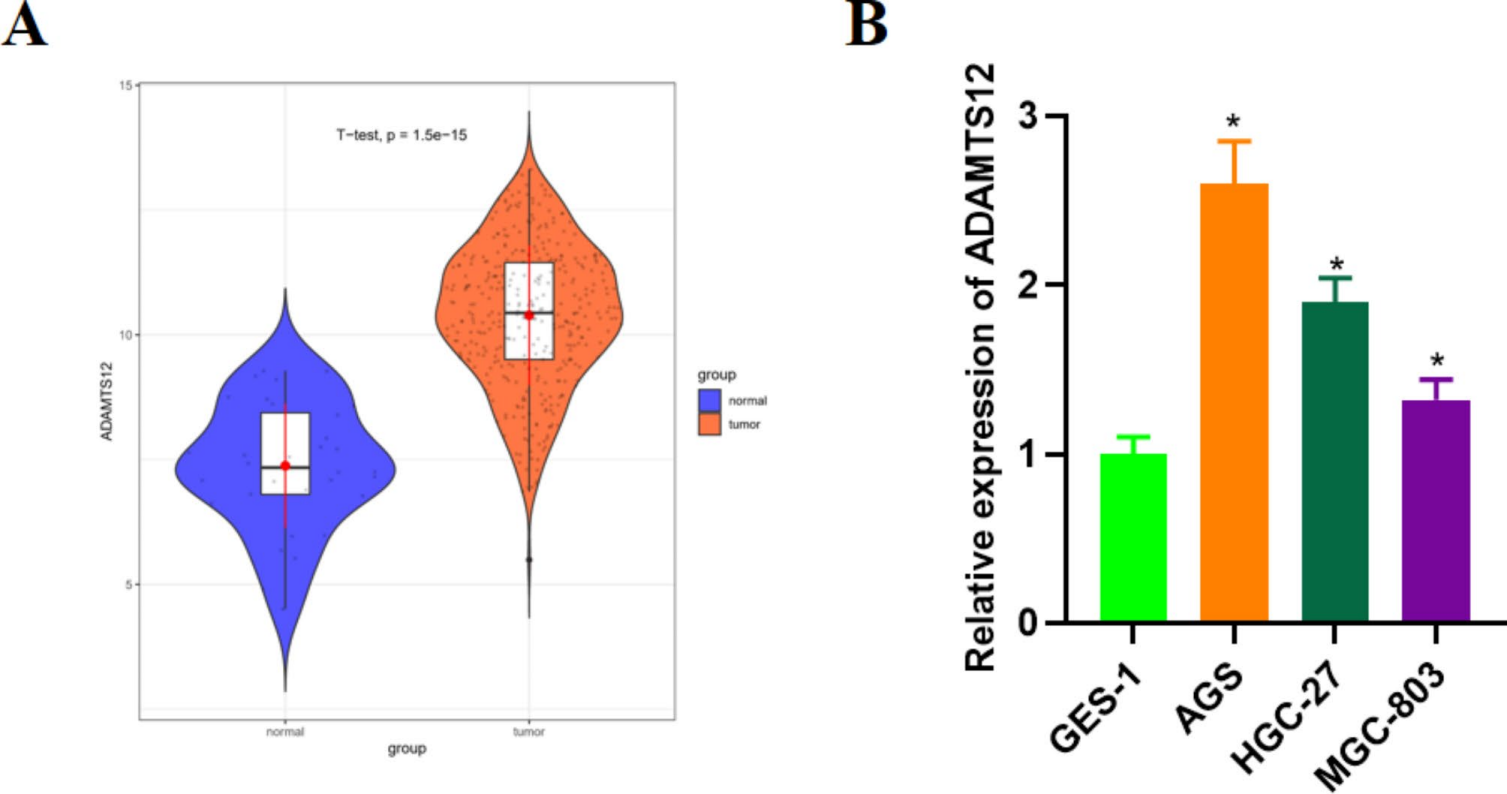
ADAMTS12 is highly expressed in GC tissues and cells. **A**: ADAMTS12 expression in GC tissues in TCGA; **B**: ADAMTS12 expression in human gastric mucosal epithelial cell line (GES-1) and human GC cell lines (AGS, HGC-27, MGC-803); * *P* < 0.05

ADAMTS12 plays an oncogenic role in the progression of multiple human cancers [21]. To explore the role of ADAMTS12 in GC progression, bioinformatics analysis was conducted. The results revealed a substantially upregulated expression level of ADAMTS12 in GC tissues (Fig. 1A). qRT-PCR revealed that ADAMTS12 was substantially upregulated in GC cells versus GSE-1 cells (Fig. 1B). These results proved that ADAMTS12 was substantially high-expressed in GC.

### ADAMTS12 enhances GC cell proliferation and glycolysis

**Fig. 2:**
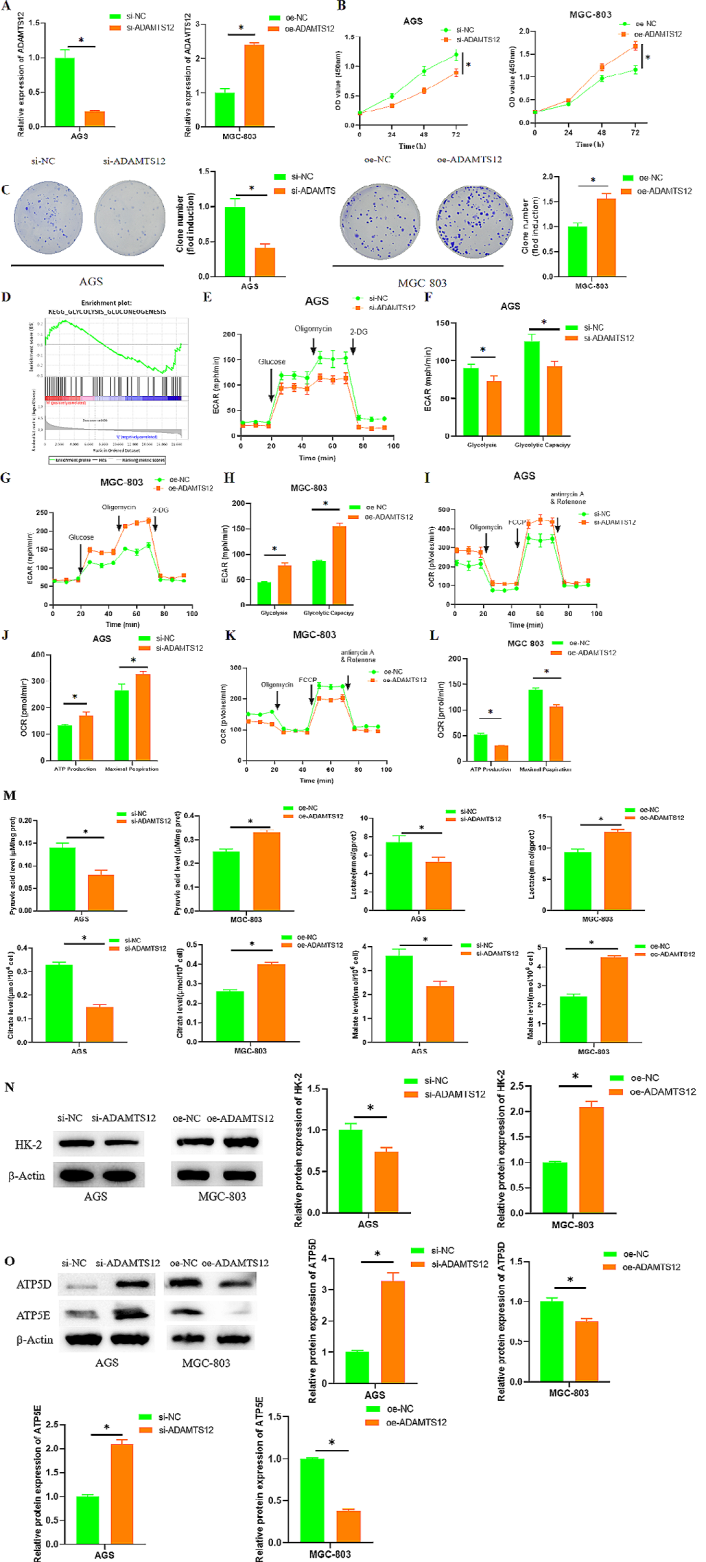
ADAMTS12 enhances GC cell proliferation and glycolysis. A: Transfection efficiency as detected by qRT-PCR; B: Cell viability after transfection as assayed by CCK-8; C: Cell proliferation after transfection as assessed via colony formation assay; D: GSEA pathway enrichment analysis reveals marked correlations of high ADAMTS12 expression with Glycolysis/Gluconeogenesis pathway; E-L: Seahorse XP 96 analysis of ECAR and OCR of GC cells in different treatment groups; M: Detection of pyruvic acid, lactate, citrate, and malate levels in GC cells in each treatment group; N-O: HK-2, ATP5D and ATP5E expression after cell transfection as tested via western blot; * P < 0.05

To probe the role of ADAMTS12 in GC progression, si-ADAMTS12 was transfected into AGS cell line expressing a relatively high ADAMTS12 level. qRT-PCR assay revealed a substantial decrease in ADAMTS12 expression after ADAMTS12 knockdown, and a significant increase in ADAMTS12 expression in MGC-803 cells after oe-ADAMTS12 transfection, indicating successful transfection (Fig. 2A). CCK-8 and colony formation assays revealed that ADAMTS12 knockdown substantially reduced cell viability and proliferative capacity (Fig. 2B-C). Additionally, ADAMTS12 was mainly enriched in the glycolysis pathway as revealed by bioinformatics analysis (Fig. 2D), and thus the relationship between ADAMTS12 and glycolysis was explored. ECAR and OCR of GC cells were tested in different treatment groups using Seahorse analyses. The glycolytic level and glycolytic capacity of GC cells were substantially reduced with ADAMTS12 knockdown, but the basal OCR and maximum OCR of GC cells were substantially increased, and overexpression of ADAMTS12 promoted the glycolytic capacity of MGC-803 cells (Fig. 2E-L). ADAMTS12 knockdown significantly hindered the production of glycolytic metabolites including pyruvic acid, lactate, citrate, and malate in GC cells, and ADAMTS12 overexpression produced the opposite effect (Fig. 2M). Western blot assay presented that ADAMTS12 knockdown substantially reduced HK-2 expression, the glycolysis-related protein, promoted the expression of ATP5D, ATP5E, the oxidative phosphorylation -related proteins (Fig. 2N-O). These data suggested that ADAMTS12 enhanced GC cell proliferation and aerobic glycolysis.

### Metformin represses GC cell proliferation and glycolysis through manipulation of ADAMTS12

**Fig. 3:**
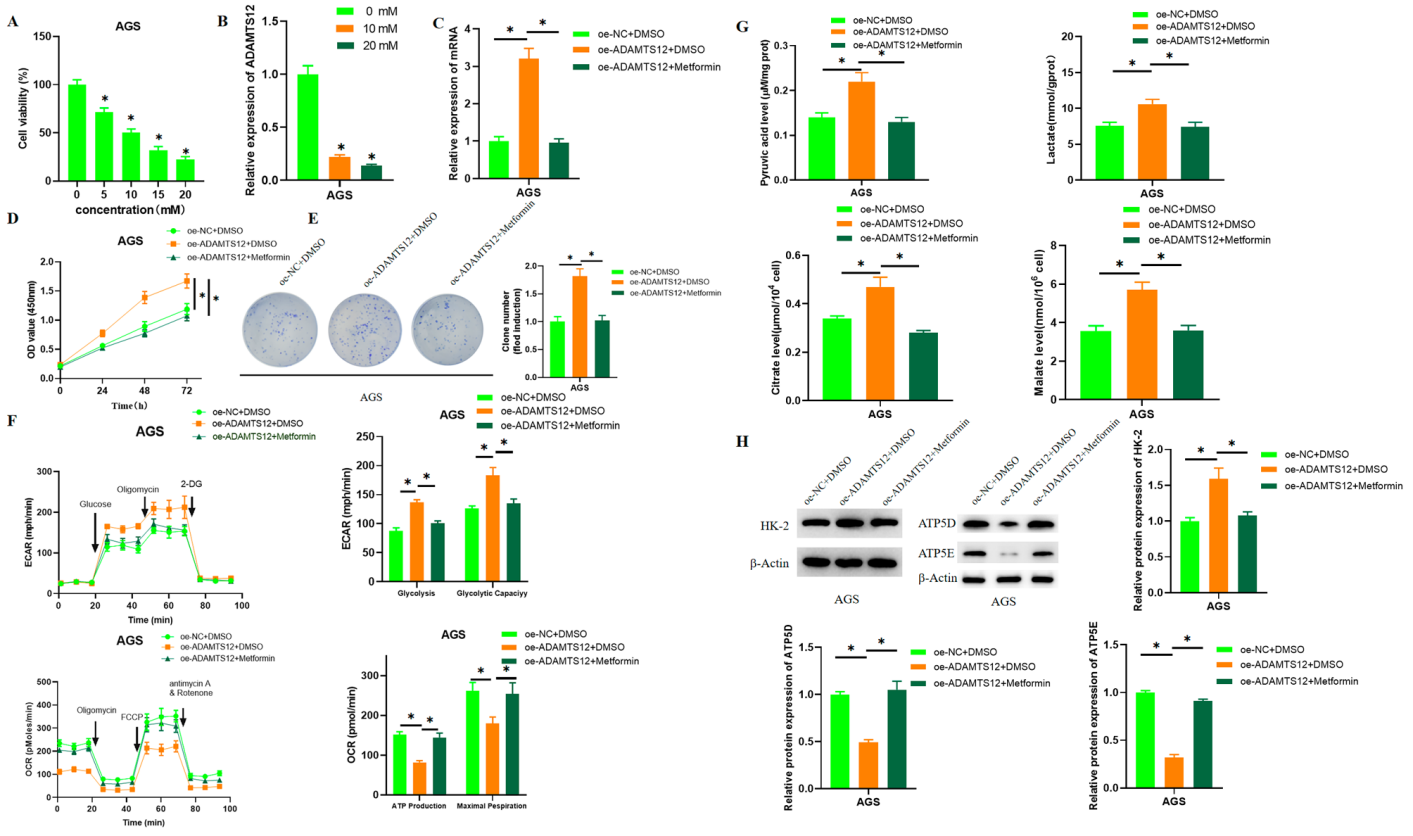
Metformin represses GC cell proliferation and glycolysis through manipulation of ADAMTS12. **A**: Cell viability after treatment with different concentrations of metformin as tested by CCK-8; **B-C**: ADAMTS12 expression after cell transfection as assayed by qRT-PCR; **D**: Cell viability after transfection as measured by CCK-8; **E**: Cell proliferation after transfection as detected via colony formation assay; **F**: Seahorse XP 96 analysis of ECAR and OCR of GC cells in different treatment groups; **G**: Detection of pyruvic acid, lactate, citrate, and malate levels in GC cells in each treatment group; H: HK-2, ATP5D and ATP5E expression after cell transfection as assayed by western blot; * *P* < 0.05

One recent study reported that metformin represses GC cell proliferation, induces cell death, and causes partial cell cycle arrest [15]. To investigate the molecular mechanism of metformin in GC, GC cells were subjected to metformin treatment of different concentrations. CCK-8 and qRT-PCR assays revealed that metformin substantially reduced cell viability and ADAMTS12 expression (Fig. 3A-B). To test our conjecture that metformin may exert its oncogenic role by downregulating ADAMTS12, cells were grouped into oe-NC + DMSO, oe-ADAMTS12 + DMSO, and oe-ADAMTS12 + Metformin. As revealed by qRT-PCR, ADAMTS12 overexpression substantially increased ADAMTS12 level, but metformin was able to attenuate the effect (Fig. 3C). CCK-8 assay presented that ADAMTS12 overexpression substantially enhanced cell viability, but this effect could be attenuated by metformin treatment (Fig. 3D). Colony formation assay revealed that ADAMTS12 overexpression notably fostered cell proliferation, but metformin treatment was able to attenuate this effect (Fig. 3E). OCR and ECAR detection revealed that in AGS cells overexpressing ADAMTS12, the glycolytic level and capacity of GC cells was noticeably increased, and the basal OCR and maximum OCR were substantially decreased. The levels of glycolytic metabolites including pyruvic acid, lactate, citrate, and malate were significantly increased. But metformin treatment was able to attenuate the stimulatory effect of ADAMTS12 overexpression on aerobic glycolytic metabolism in AGS cells (Fig. 3F-G). Western blot results indicated that ADAMTS12 overexpression was able to increase HK-2 expression and decrease ATP5D, ATP5E protein expression, but metformin attenuated this effect (Fig. 3H). In short, these demonstrated that metformin repressed GC cell proliferation and glycolysis by manipulating ADAMTS12.

### Metformin suppresses GC tumor growth in vivo by modulating ADAMTS12

**Fig. 4:**
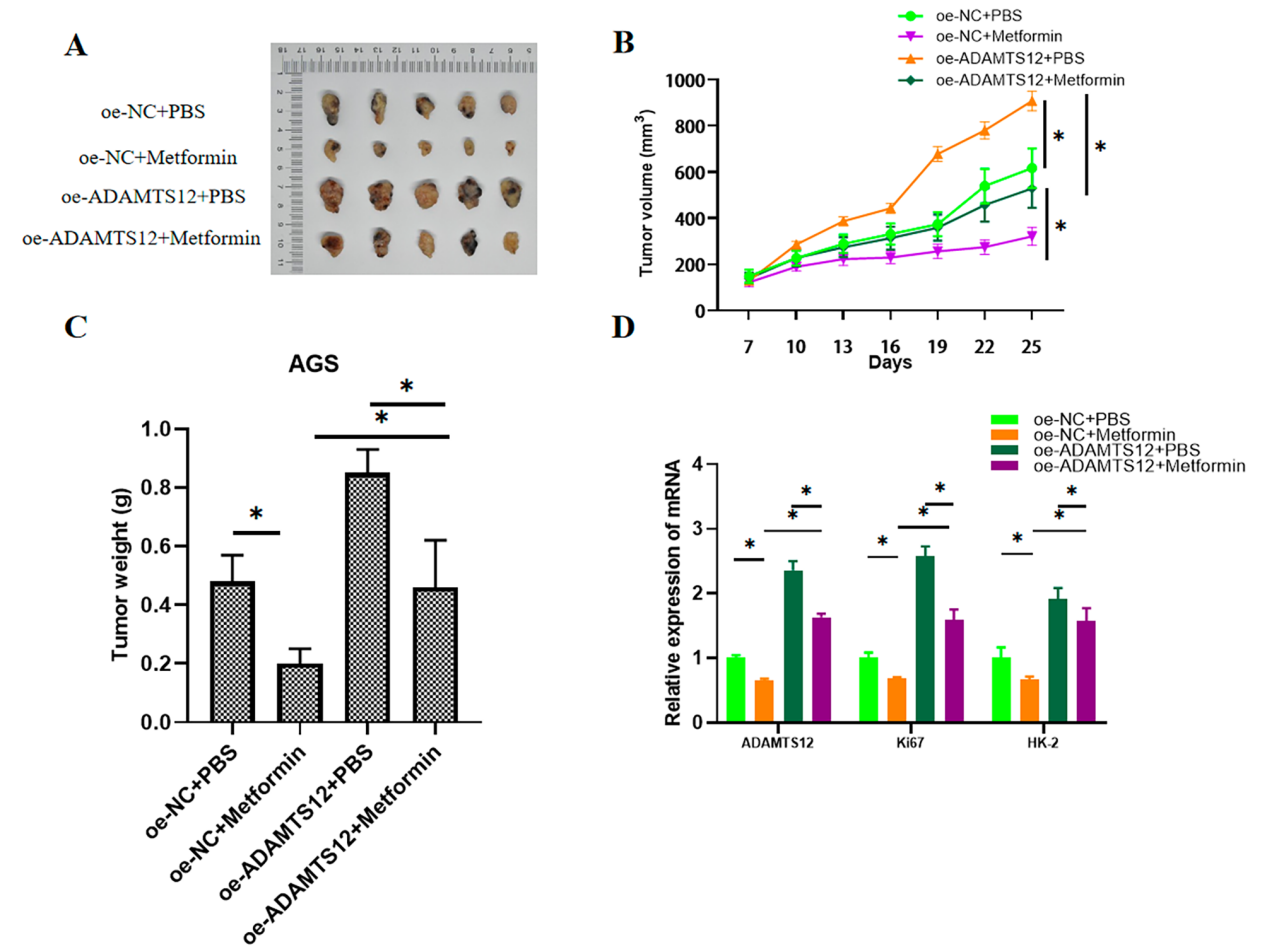
Metformin suppresses GC tumor growth in vivo by modulating ADAMTS12. **A-C**: Tumor volume and weight; **D**: ADAMTS12, ki67, and HK-2 expression in tumor tissues as detected by qRT-PCR; * *P* < 0.05

To confirm molecular mechanism of metformin in GC progression and its dependence on ADAMTS12, AGS cells treated with oe-NC or oe-ADAMTS12 were injected into mice, which were treated with metformin or PBS. Compared with the oe-NC + PBS group, ADAMTS12 overexpression accelerated tumor growth and increased tumor volume and weight, and treatment with metformin alone restrained tumor growth. But the concomitant treatment of metformin and ADAMTS12 overexpression slowed the stimulatory effects on tumor growth, volume, and weight (Fig. 4A-C). These findings suggested that ADAMTS12 overexpression significantly facilitated tumor growth, and such promotion was effectively attenuated by metformin treatment. Finally, qRT-PCR analysis presented that compared with the oe-NC + PBS group, ADAMTS12 overexpression increased expression levels of ADAMTS12, Ki67, and HK-2, but metformin treatment effectively attenuated the effects (Fig. 4D). In conclusion, these data confirmed that metformin repressed GC tumor growth in vivo by modulating ADAMTS12.

## Discussion

GC is the third largest reason for cancer-related death worldwide [22]. Although there have been various improvements in the management of GC, the current situation on prognosis of GC patients remains unsatisfactory due to tumor metastasis and recurrence. Therefore, it is urgent to find new targets for GC diagnosis and treatment [23]. ADAMTS12 has been found to be associated with the malignant behaviors of tumor cells [24]. In pancreatic cancer, ADAMTS12 facilitated cell migration and epithelial-mesenchymal transition and indicates poor prognosis [25]. I human choriocarcinoma, ADAMTS12 promotes cell-extracellular matrix adhesion and invasion by a αvβ3 integrin heterodimer related mechanism [26]. In addition, ADAMTS12 is highly expressed in GC and responsible for tumor microenvironment (TME) status and tumor energy metabolism and transformation, acting as a TME-related cancer promoter [27]. In the current study, ADAMTS12 was highly expressed in GC, agreeing with previous studies. In addition, ADAMTS12 was confirmed to promote the proliferation and glycolysis of GC cells. The present study showed for the first time the role of ADAMTS12 in glycolysis of GC cells, indicating that ADAMTS12 might be a novel target for GC management.

Metformin is a widely prescribed oral antidiabetic agent, and recently a growing number of studies have reported its potential antitumor effects in different cancer types [28–31]. For example, Schuler et al. [32] revealed that metformin can reduce tumor proliferation by inhibiting mTOR pathway in patients with endometrial cancer. Chen et al. [33] proved that metformin can hinder the growth of AR-negative prostate cancer by modulating AMPK/autophagy signaling. DPP4 is involved in immune cell infiltration within the TME, and metformin is able to inhibit Hek293 cell growth by increasing DPP4 expression [34]. In the current study, we exhibited that metformin could suppress the expression of ADAMTS12 and inhibit the proliferation and glycolysis of GC cells. Further cellular and mice experiments confirmed that metformin could reduce the promoting influence of ADAMTS12 overexpression on the proliferation and glycolysis of GC cells. Taken together, this study proposed the potential mechanism of metformin’s antitumor role in GC.

For the first time, this study presented that metformin could inhibit the proliferation and glycolysis of GC cells through modulatingADAMTS12, which deepens the understanding of the mechanism and anti-cancer potential of metformin in treating GC. However, there are still some shortcomings in our study. For example, it lacks clinical tests to verify the conclusion that metformin inhibits GC via ADAMTS12. Therefore, clinical samples will be collected to explore the mechanism that metformin targets ADAMTS12 to inhibit GC. In conclusion, our study deepens the understanding of the molecular mechanism by which metformin inhibits GC progression. These findings suggest the potential of metformin in treating GC and the possibility of ADAMTS12 being a novel target for GC treatment.

## Data Availability

The data used to support the findings of this study are included within the article.
